# Prioritizing candidate genes post-GWAS using multiple sources of data for mastitis resistance in dairy cattle

**DOI:** 10.1186/s12864-018-5050-x

**Published:** 2018-09-06

**Authors:** Zexi Cai, Bernt Guldbrandtsen, Mogens Sandø Lund, Goutam Sahana

**Affiliations:** Center for Quantitative Genetics and Genomics, Department of Molecular Biology and Genetics, Aarhus University, 8830 Tjele, Denmark

**Keywords:** Dairy cattle, Mastitis, Post-GWAS, Gene-base analysis, RNA-seq

## Abstract

**Background:**

Improving resistance to mastitis, one of the costliest diseases in dairy production, has become an important objective in dairy cattle breeding. However, mastitis resistance is influenced by many genes involved in multiple processes, including the response to infection, inflammation, and post-infection healing. Low genetic heritability, environmental variations, and farm management differences further complicate the identification of links between genetic variants and mastitis resistance. Consequently, studies of the genetics of variation in mastitis resistance in dairy cattle lack agreement about the responsible genes.

**Results:**

We associated 15,552,968 imputed whole-genome sequencing markers for 5147 Nordic Holstein cattle with mastitis resistance in a genome-wide association study (GWAS). Next, we augmented *P*-values for markers in genes in the associated regions using Gene Ontology terms, Kyoto Encyclopedia of Genes and Genomes pathway analysis, and mammalian phenotype database. To confirm results of gene-based analyses, we used gene expression data from *E. coli*-challenged cow udders. We identified 22 independent quantitative trait loci (QTL) that collectively explained 14% of the variance in breeding values for resistance to clinical mastitis (CM). Using association test statistics with multiple pieces of independent information on gene function and differential expression during bacterial infection, we suggested putative causal genes with biological relevance for 12 QTL affecting resistance to CM in dairy cattle.

**Conclusion:**

Combining information on the nearest positional genes, gene-based analyses, and differential gene expression data from RNA-seq, we identified putative causal genes (candidate genes with biological evidence) in QTL for mastitis resistance in Nordic Holstein cattle. The same strategy can be applied for other traits.

**Electronic supplementary material:**

The online version of this article (10.1186/s12864-018-5050-x) contains supplementary material, which is available to authorized users.

## Background

Mastitis is the commonest and costliest disease in the dairy cattle industry [1], decreasing milk production and quality, incurring treatment costs for farmers, and negatively affecting animal welfare. Mastitis incidence, severity, and outcomes are influenced by several factors, including the pathogen type, host immunity, and environment [2]. On the host side, mastitis resistance, cow age, and lactation stage influence mastitis risk [3]. Although genetics plays a role in mastitis resistance, the trait has low heritability [4, 5]. Nonetheless, breeding can help to reduce the incidence of mastitis. In this regard, identifying candidate genes affecting mastitis resistance may help in breeding cows for improved udder health, especially in transferring information across breeds for genomic prediction [6].

Genome-wide association studies (GWASs) are widely used to find DNA variants associated with complex traits like mastitis [7]. Previous studies have identified quantitative trait loci (QTL) that are associated with variations in CM and its indicator trait, somatic cell count (SCC) [8–11]. These studies have proposed candidate genes, such as *GC*, *NPFFR2* [12], *TRAPPC*, *ARHGAP39* [13], *LY6K*, *LY6D*, *LYNX1*, *LYPD2*, *SLURP1*, and *PSCA* [14]. However, concordance among these studies is low, suggesting difficulty in identifying reliable candidate genes for mastitis. New approaches integrating additional information with GWAS results can help with this problem. For example, a gene-based association statistical approach accounts for the correlation structure among single-nucleotide polymorphisms (SNPs) within the gene and may have higher power to prioritize genes within an identified region to be candidates [15]. This approach can be especially helpful when the lead SNP from association analysis is not the causal mutation or is not located within the causal gene. We expect that the causal gene will be enriched in trait-associated SNPs compared to neighboring genes.

Various approaches for gene-based analysis have been implemented in programs like GCTA [16], MAGMA [17], GATES [18], VEGAS [19], and so on [20]. As implemented in MAGMA [17], gene-based analysis uses a multiple regression model to test the joint effect of multiple markers from a gene and is efficient in finding candidate genes [17]. Gene expression data provide another source of information to identify candidate genes. The most powerful method of incorporating RNA-seq data is expression quantitative trait locus (eQTL) mapping, which can serve as tool for identifying genetic variants that affect gene regulation [21]. In a multitrait setting, eQTL mapping can help in distinguishing between pleiotropic and linkage effects [22]. However, eQTL studies remain expensive, impeding their application to livestock. As an alternative, specifically designed gene expression studies (RNA-seq) in target tissues from healthy and affected individuals can be used as biological evidence post GWAS to prioritize candidate genes [23].

We hypothesized that combining gene-based association statistics, gene annotation, and gene expression, along with known phenotypes related to the genes, would enable us to prioritize candidate genes for quantitative traits. In this study, we performed GWAS using 15,552,968 imputed markers on 5147 Nordic Holstein cattle for resistance to CM. Post GWAS, we studied candidate genes for gene-based association signals, and made annotations using the Gene Ontology (GO) database [24], Kyoto Encyclopedia of Genes and Genomes (KEGG) pathway analysis [25], and mammalian phenotype databases [26]. We confirmed results of gene-based association strength with mammary gland expression during *E. coli* challenge experiments [23]. Variant annotations were used to investigate candidate mutations within the prioritized candidate genes.

## Results and discussion

### GWAS for resistance to clinical mastitis in Nordic Holstein cattle

**Fig. 1:**
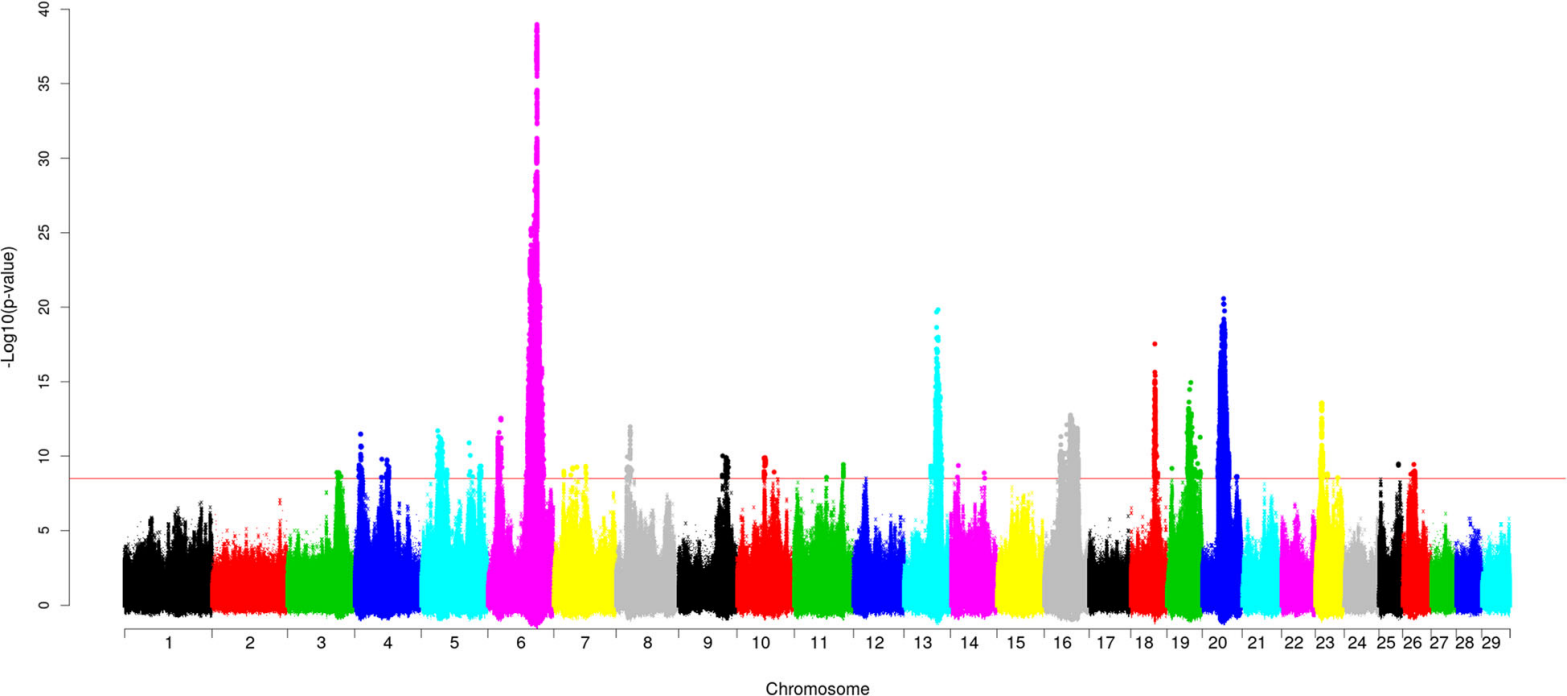
Manhattan plot for association of SNPs with resistance to clinical mastitis of Nordic Holstein cattle. Red horizontal line indicates genome-wide significance level [−log_10_(P) = 8.5]. Base positions are given as position in UMD 3.1.1 [59] bovine genome assembly

Using our previously described GWAS approach [27], we ran an association analysis with imputed whole-genome sequencing (WGS) variants for resistance to CM in Nordic Holstein cattle. Figure 1 presents the Manhattan plot of the GWAS results. Table 1 lists the lead SNPs (i.e., most significantly associated SNPs) and positional candidate genes (i.e., annotated genes closest to lead SNPs).

**Table 1:** Genomic regions identified by genome-wide association analysis of resistance to clinical mastitis in Nordic Holstein cattle

BTA	Base position	Effect	–log_10_(p)	Region^***^	Gene	Annotation
3	92,927,352	−1.34	8.88	91,961,838~ 93,178,041	*ENSBTAG00000010814* (28,361 bp)	intergenic
4	10,928,348	−2.39	11.48	10,113,846 ~ 11,178,507	*TFPI2* (107,131 bp)	intergenic
4	58490979^*^	1.44	9.44	57,757,451 ~ 58,741,047	*ENSBTAG00000020620* (233,286 bp)	intergenic
5	30,211,323	−1.54	11.71	29,850,270 ~ 30,461,626	*BCDIN3* (599 bp)	upstream
5	106371995^*^	− 1.52	9.23	105,444,242 ~ 106,622,012	*CCND2* (95,176 bp)	intergenic
6	88,729,872	2.78	38.97	88,479,895 ~ 88,980,376	*GC*	intron
6	23469606*	1.63	12.53	23,219,637 ~ 23,719,758	*MANBA*	intron
6	93131207^**^	2.05	12.63	92,131,530 ~ 93,382,644	*CCDC158* (72,869 bp)	intergenic
7	57,794,761	1.36	9.32	57,545,197 ~ 58,044,816	*ENSBTAG00000019739* (33,697)	intergenic
8	25,684,799	1.83	11.98	25,434,812 ~ 25,935,079	*ADAMTSL1*	intron
9	80,007,099	−1.45	10.02	79,238,565 ~ 80,257,157	*NMBR* (604,558 bp)	intergenic
10	51,191,670	−1.63	9.90	50,733,332 ~ 51,441,800	*MYO1E*	intron
11	88,742,878	1.47	9.43	88,150,188 ~ 88,993,125	*ID2*	intergenic
13	62,017,506	−1.95	19.82	61,295,534 ~ 62,267,717	*PDRG1* (2545 bp)	upstream
14	61,344,981	1.76	8.88	61,020,081 ~ 61,594,984	*ZFPM2*	intron
16	47,836,093	−1.88	12.77	47,048,599 ~ 48,086,099	*ACOT7*	intron
18	43,909,571	2.38	17.52	43,659,734 ~ 44,159,716	*ENSBTAG00000004994* (17,651 bp)	intergenic
19	43,038,655	−1.73	14.95	42,148,461 ~ 43,288,858	*STAT5A*	intron
20	38,471,456	−2.88	20.57	38,221,493 ~ 38,721,830	*SPEF2*	intron
23	11,477,905	1.53	13.58	11,204,757 ~ 11,727,945	*MDGA1* (52,973 bp)	intergenic
25	35,354,412	1.46	9.47	35,104,498 ~ 35,604,430	*CUX1*	intron
26	20,463,679	1.41	9.44	20,214,011 ~ 20,713,741	*SLC25A28* (2425 bp)	downstream

^*^The lead SNP was found in the second round

^**^The lead SNP was found in the third round

^***^The method to define the QTL interval can be found in Method

We observed 22 association signals across the genome (−log_10_(P) = 8.5), which explained 14% of variance among de-regressed breeding values for resistance to CM. The strongest association signal was on *Bos taurus* autosome (BTA) 6, where the lead SNP was BTA6:88729872 (rs109803407) located within *GC*. The *GC* and nearby *NPFFR2* genes were previously proposed as candidate genes for mastitis in cattle [12]. The second lead SNP on this chromosome was BTA6:23469606 (rs380325826), located within the intron of *MANBA*. According to Uniport [28], this gene encodes a protein involved in the N-glycan degradation pathway, but there is no known biological function relating this gene to mastitis resistance. A third association signal on BTA6 was observed at BTA6:93131207 (rs379964407), located close to a lead SNP from a previous study (BTA6:93381472, rs41655339) [29]. The lead SNP on BTA20, BTA20:38471456 (rs472814468), was located within a previously reported QTL [12] upstream of *SPEF2*, which is related to the GO term “immune system development”. The lead SNP on BTA13 was BTA13:62017506 (rs211080099), located close to a previously reported QTL for CM in Norwegian Red cattle [30]. The gene closest to this SNP was *PDRG1*. The lead SNP on BTA18, BTA18:43909571 (rs464881101), was located near *ENSBTAG00000004994* (novel gene). The lead SNP on BTA19 was BTA19:43038655 (rs134993207), located near *STAT5A*. Previous research proposed this gene as a candidate gene for cattle mastitis [31, 32]. This lead SNP was also near *BRCA1* (BTA19:43714457 ~ 43,783,351), which was previously proposed as a candidate gene for mastitis resistance [33]. Other lead SNPs on chromosomes 4, 16, and 20 are located near previously reported QTL for CM or SCC [12, 13, 34].

### Identification of candidate genes by gene-based association statistics

We performed a gene-based association statistical approach using MAGMA [17] to search for candidate genes. Due to long-range linkage disequilibrium (LD) in the cattle genome, several genes in some QTL regions showed significant signals (Additional file 1: Table S1); therefore, we considered the top five genes from each association signal (Table 2). Next, we included information from the GO database [24], KEGG pathway analysis [25], and the mammalian phenotype databases [26] to narrow the candidate gene list. We searched these databases for the top five genes from the gene-based analysis (Table 2) and for genes closest to the lead SNPs (Table 1), if not already included in the previous list. Enrichment analysis using the clusterProfiler package [35] did not show any enrichment for GO terms; however, the inclusion of some GO terms involved in mastitis will be useful for picking candidate genes with biological support.

**Table 2:** Top five genes based on gene-based association statistics for resistance to clinical mastitis

Lead SNP	Top 5 Genes^*^	Gene *P* value
3:92927352	*PLPP3, TXNDC12, RAB3B, NRDC, ENSBTAG00000046915*	1.46e-08
4:10928348	*ASB4, ANKIB, COL28A1, DYNC1I1, PON1*	1.83e-06
4:58490979	*TBX20*, *IMMP2L*, *ST7*, *LAMB4, ELMO1*	9.77e-09
5:30211323	*SLC2A13*, *SLC38A1*, *RPAP3, TMEM117*, *LIMA1*	6.80e-10
5:106360448	*CCDC77,* LOC100336690*,ABCC9,KDM5A*, *FGF6*	1.56e-08
6:23469606	*TBCK, TSPAN5, PPA2, PPP3CA, NFKB1*	1.33e-09
6:87299659	*NPFFR2, SULT1E1, SLC4A4, DCK, LOC100140490*	2.50e-26
6: 93131207	*PARM1, SHROOM3, MTHFD2L, SEPT11, EPGN*	2.16e-14
7:31253987	*C3, PCDHB11, PCDHB6, FBN3, LOC786512*	1.27e-08
8:25684799	*ADAMTSL1, MLLT3, PLPPR1, MTAP, FOCAD*	3.15e-08
9:80007099	*ESR1, RAET1G, PPP1R14C, SYNE1, SHPRH*	4.16e-07
10:51191670	*RORA, MYO1E, RNF111, DUOX1,SLC28A2*	2.93e-08
11:88742878	*PDIA6, ATP6V1C2, CAPG, TBC1D8, MGAT4A*	4.43e-08
13:62017506	*BMP7, TFAP2C, NSFL1C, NDRG3, REM1*	2.01e-14
14:61344981	*DPYS, KCNQ3, TPD52, RSPO2, PARP10*	1.44e-07
16:47836093	*SMYD3, ACOT7, MEGF6, ENSBTAG00000019339, CHD5*	8.28e-13
18:43909571	*HPN, GPI, WTIP, PDCD2L, TGFB1*	7.08e-09
19:43038655	*SP2, TBCD, OSBPL7, SRCIN1, CBX1*	2.22e-13
20:38471456	*PLCXD3, WDR70, RICTOR, MROH2B, NUP155*	2.99e-14
23:11477905	*ZFAND3, CPNE5, DNAH8, KCTD20, PPARD*	1.05e-11
25:35354412	*TMEM130, CORO7, VASN, CDIP1, DNAJA3*	2.09e-08
26:20463679	*PDE6C, BTRC, SEMA4G, CNNM2, SLK*	7.43e-09

^*^Top five genes selected based on the ranking of *P* value, if the –log_10_ (*P*) > 5.60, the genes are listed in the table

^#^ The *P* value listed in the table is for the gene with highest *P* value among the top five genes showing association. The model to calculate the gene *P* value in MAGMA [17] was snp-wise = mean

As GO terms for mastitis-related biological pathways, we chose “inflammatory response” and all terms related to “immune” and “wound healing”. Ten candidate genes for mastitis resistance (Tables 1 and 2) were associated with these GO terms: *PLPP3* (canonical Wnt signaling pathway involved in positive regulation of wound healing), *NFKB1* (inflammatory response, innate immune response), *C3* (inflammatory response), *ESR1* (regulation of inflammatory response), *RORA* (negative regulation of inflammatory response), *TGFB1* (inflammatory response, response to wounding, immune response based on somatic recombination of immune receptors built from immunoglobulin superfamily domains), *STAT5A* (positive regulation of inflammatory response), *RICTOR* (regulation of inflammatory response), *SPEF2* (immune system development), and *PPARD* (wound healing)*.* From this list, *STAT5A* is near the lead SNP on BTA19 (Table 1) and is associated with the GO term “positive regulation of inflammatory response”. *SPEF2*, related to “immune system development,” harbors the lead SNP on BTA20 (Table 1).

The KEGG pathway analysis showed that *ESR1*, *CCND2, NFKB1*, and *STAT5A* are involved in the signaling of prolactin, a polypeptide hormone that is involved in a wide range of biological functions including immunomodulation. *CCND2* is located near the second lead SNP on BTA5 (Table 1). *CCND2*, *FGF6*, and *NFKB1* are part of the PI3K-Akt signaling pathway that is an component of innate immunity [36]. *NFKB1*, *FGF6, PPP3CA*, and *TGFB1* belong to the MAPK signaling pathway. Some MAPKKKs may activate ERK1/2 in response to pro-inflammatory stimuli.

The mammalian phenotype database [26] provided useful phenotypic information about several genes. A mutation in *ELMO1* could cause a decrease in natural killer T-cell numbers. Some mutations in *PPP3CA* could cause abnormal T-cell proliferation. Mutations in *DCK* could cause abnormal T-cell differentiation and decreased response to antigen. Mutations in *GC* and *ID2* could cause abnormal immune system physiology. *ID2* harbors the lead SNP on BTA11 (Table 1). Mutations in *STAT5A* can cause increased mammary gland apoptosis, abnormal mammary gland physiology, and decreased T-cell proliferation. Mutations in *CUX1* can cause abnormal T-cell differentiation, abnormal tumor necrosis factor levels, and small thymus. The lead SNP on BTA25 is a variant in the intron of *CUX1*. Mutations in *IMMP2L* could cause negative or positive T-cell selection. Mutations in *NFKB1* could cause abnormal inflammatory or humoral immune responses. Mutations in *NPFFR2* could cause abnormal macrophage activation, which is involved in the immune response. Mutations in *C3* and *TGFB1* could cause abnormal immune system physiology and inflammatory response. Mutations in *ESR1* could cause abnormal immunoglobulin levels. Mutations in *RORA* could cause abnormal immune system physiology. Mutations in *CORO7* could cause enhanced wound healing. Mutations in *SLK* could cause abnormal immune cell physiology and abnormal T cell activation.

### Combined analysis of RNA-seq data with gene-based association statistics

To prioritize candidate genes or choose other candidate genes (in addition to the top five genes from gene-based association and genes closest to the lead SNPs), we confirmed the significant genes from genetic analysis using differentially expressed genes (DEGs) in udders from an *E. coli* challenge experiment [23]. We identified 115 genes that were significant in gene-based association analysis and were DEGs in RNA-seq data. Table 3 gives the top gene for each QTL, and Additional file 1: Table S2 gives the full gene list.

**Table 3:** Top genes from combined analysis of gene-based association statistics and differential gene expression in udders for each QTL

Gene	Location	Gene P value	RNA-seq FDR^#^	Putative function	Differential expression^##^
*CYP4X1*	BTA3: 99666161~ 99,714,764	1.42e-7	3.47e-4	MP: abnormal humoral immune response	Down
*AOAH*	BTA4:60940663~ 61,125,662	1.51e-8	1.66e-11	GO: negative regulation of inflammatory response	Up
*ENSBTAG00000032429*	BTA5: 32263608~ 32,264,561	1.02e-9	4.71e-2	NA	Down
*SLC6A12*	BTA5: 107649324~ 107,666,752	1.14e-7	1.03e-3	NA	Up
*NFKB1*	BTA6:23557311~ 23,679,508	1.33e-9	4.02e-2	GO: inflammatory response, innate immune response	Up
*CSN1S2*	BTA6: 87262457~ 87,280,936	2.60e-17	7.65e-3	KEGG: Prolactin signaling pathway	Down
*SHROOM3*	BTA6: 93340874~ 93,398,475	1.83e-20	1.67e-2	NA	Down
*FBN3*	BTA7:18006365 ~ 18,076,590	1.13e-8	5.92e-10	NA	Down
*TAGAP*	BTA9: 96806294~ 96,815,764	2.09e-6	2.38e-3	NA	Up
*SLC28A2*	BTA10: 65395125~ 65,427,753	2.93e-8	2.75e-3	NA	Up
*PDIA6*	BTA11:86834898~ 86,857,648	3.56e-10	3.58e-2	NA	Up
*HCK*	BTA13: 62106257~ 62,151,619	1.48e-12	3.13e-22	GO: innate immune response	Up
*ACOT7*	BTA16: 47827124~ 47,934,930	6.66e-14	2.83e-2	NA	Up
*HPN*	BTA18:45971859~ 45,991,833	3.50e-11	4.53e-2	NA	Down
*PLEKHH3*	BTA19: 43366278~ 43,374,706	6.46e-13	1.73e-3	NA	Down
*MROH2B*	BTA20: 33456349~ 33,528,569	5.00e-15	8.62e-5	MP: abnormal T cell physiology and decreased T cell proliferation	Down
*ENSBTAG00000010730*	BTA23: 10627244~ 10,628,260	5.98e-9	5.36e-6	NA	Up
*CDIP1*	BTA25: 3650394~ 3,673,924	2.83e-9	1.27e-2	NA	Down
*CRTAC1*	BTA26: 18869719~ 19,013,761	8.49e-9	3.72e-2	NA	Down

^#^Results from Fang et al. [23]

^##^Comparison between infected mammary glands and controls at 24 h post intra-mammary infection with *E. coli*

Joint analysis suggested three candidate genes on BTA3, and the mammalian phenotype database showed that a mutation in *CYP4X1* could cause abnormal humoral immune response in mice. On BTA4, joint analysis suggested *AOAH* as a candidate gene, which was linked to the GO term “inflammatory response”. Joint analysis identified 13 candidate genes on BTA5, including *LALBA* (BTA5:30211323, rs41655922) with GO term “defense response to Gram-positive bacterium”. On BTA6, joint analysis uncovered 18 candidate genes, eight with additional support: *NFKB1* (BTA6:23469606, rs380325826), *PDGFRA* (BTA6:87299659, rs383420156), *CSN1S2* (BTA6:87299659, rs383420156), *CSN2* (BTA6:87299659, rs383420156), *PF4* (BTA6:88729872, rs109803407), *CXCL2* (BTA6:88729872, rs109803407), *EREG* (BTA6:93131213), and *CXCL13* (BTA6:93131213). *NFKB1*, located at BTA6:23557311–23,679,508, is involved in “inflammatory response” and “innate immune response”. This gene is near the second association signal on BTA6 (Table 1). *CSN1S2* is associated with “defense response to bacterium” and “inflammatory response”. *CSN2* belongs to the prolactin signaling pathway. *CXCL2* and *CXCL13* are associated with “immune response”. *PF4* is associated with “inflammatory response” and “immune response”. *PDGFRA* belongs to the PI3K-Akt and MAPK signaling pathways. *EREG* has the GO term “positive regulation of innate immune response”.

On BTA7, we found that *VAV1* has additional support and may be involved in immune responses [37]. On BTA11, we found *IL1R2* and *IL1RL1* with additional support, which have GO terms “involved in inflammatory response” and “positive regulation of inflammatory response”, respectively. On BTA13, we found five genes with additional support: *ANGPT4*, *HCK*, *CCM2L*, *BPIFA1*, and *BPIFB1*. *ANGPT4* is involved in the Ras and PI3K-Akt signaling pathways, *HCK* plays an important role in innate immunity [38], and *CCM2L* mediates wound healing. *HCK*, *BPIFA1*, and *BPIFB1* all have the GO term “innate immune response”.

On BTA16, we identified *SELP*, *TNFRSF18*, and *ISG15* as candidate genes with additional support. *SELP* and *TNFRSF18* are associated with “inflammatory response”, and *TNFRSF18* has the GO term “immune response”. *ISG15* is associated with “defense response to bacterium”. On BTA18, we found a gene involved in biosynthesis of antibiotics, *BCKDHA*. On BTA19, *TRPV2*, *DHX58*, *STAT3*, and *ITGB3* stood out as candidate genes with additional support. *TRPV2* is involved in inflammatory mediator regulation of TRP channels. *DHX58* has the GO term “negative regulation of innate immune response”. *STAT3* is involved in the prolactin signaling pathway. According to the mammalian phenotype database a mutation in this gene in mice could cause abnormal innate immunity and increased susceptibility to bacterial infection. *ITGB3* has the GO term “wound healing”.

On BTA20, *FYB1* is a possible candidate gene with additional support and is involved in immune response [39]. On BTA23, *F13A1* and *PLA2G7* are candidate genes with additional support. *F13A1* is involved in complement and coagulation cascades. The mammalian phenotype database associated a mutation in *PLA2G7* with increased susceptibility to bacterial infection. On BTA25, *ORAI2* stood out. According to the mammalian phenotype database, mutations in *ORAI2* could cause abnormal T-cell activation and physiology. On BTA26, we found *SCD* with additional support. Mutation in this gene could cause increased susceptibility to bacterial infection from mammalian phenotype database.

### Variant annotations to help find candidate genes and mutations

**Fig. 2:**
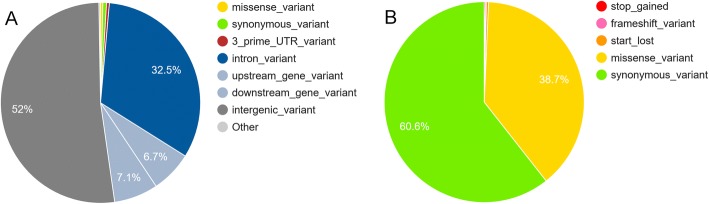
Distribution of VEP annotations for SNPs in LD (r^2^ > 0.2) with lead SNPs. **a** Percentage of each annotation category among all SNPs within LD with lead SNPs. **b** Proportion of each annotation among variants that can change protein sequences

In cattle, long-range LD can result in wide QTL regions [40]. Therefore, causative variants may be in the LD region of the lead SNP, rather than in genes harboring the lead SNP. To identify additional candidate genes, we annotated all SNPs in LD (r^2^ > 0.2) with the lead SNP using the Variant Effect Predictor (VEP) [41]. We annotated 61,992 SNPs, which were typically intergenic followed by intronic variants (Fig. 2a). Among variants located within coding sequences (Fig. 2b), most SNPs were synonymous variants followed by missense variants. We identified 136 genes with at least one missense, start-loss, stop-gain, frameshift, splice-donor, or splice-acceptor variant. Among them, 16 genes were in the previously identified candidate gene sets (i.e., top five genes from gene analysis, nearest genes, and joint analysis; Tables 1-2 and Additional file 1: Table S1‑S2). These genes were: *FAM151A, SLC6A12, NFKB1, NPFFR2, PDIA6, CASS4, BPIFA1, BPIFB1, DCSTAMP, KRT24, KCNH4, PLEKHH3, SPEF2, CRTAC1, SCD*, and *ENSBTAG00000006539*.

### Putative causal genes for resistance to clinic mastitis

**Fig. 3:**
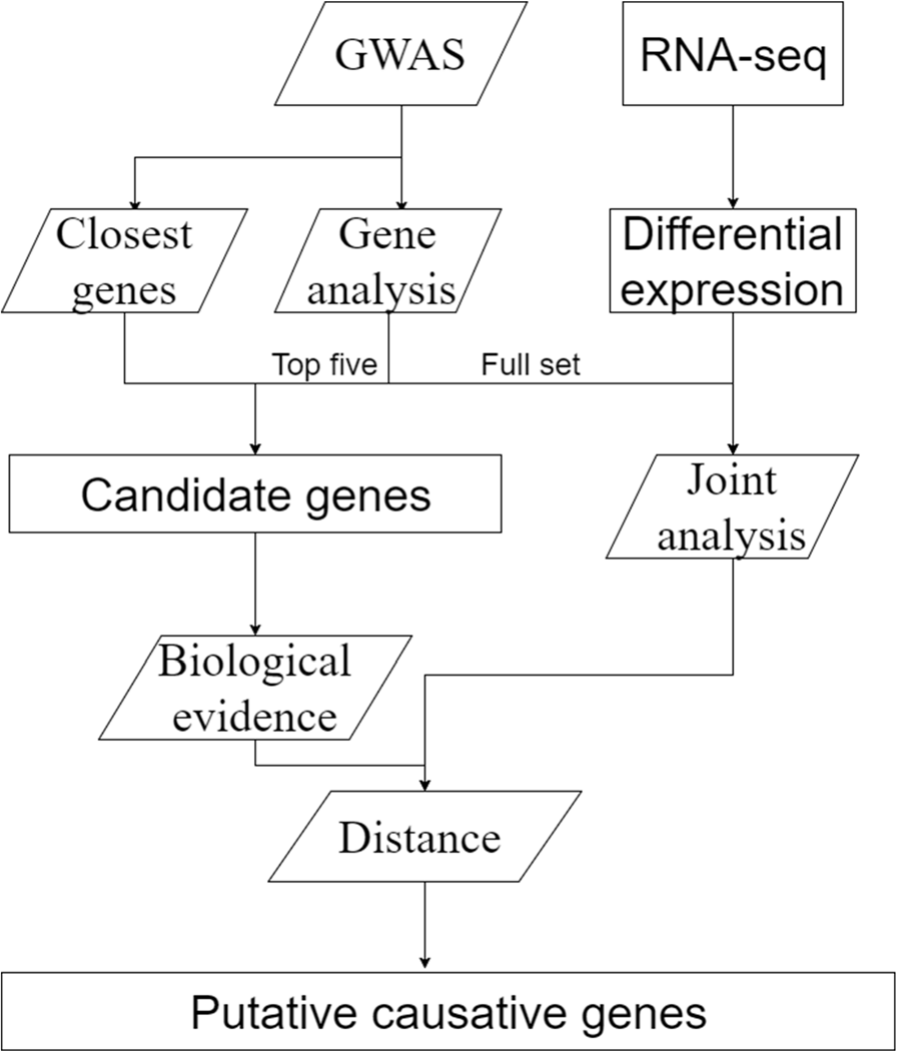
Flow chart of procedure to find putative causal genes. Parallelogram means analysis, rectangle means output, and text above arrow indicates which part of result to use

The three analyses described above helped us to select putative causal genes from among the candidate genes (summarized in Table 4). Although our analysis (Fig. 3) was effective, we still had some QTL without any good hits. We examined the flanking regions of these QTL to uncover the reason for the lack of putative causal genes and to pick some genes for future study. No genes were recognized in the region around the lead SNP in BTA8 (BTA8:25684799, rs378067069). However, the lead SNP was located is in the intron of *ADAMTSL1* (BTA8: 25,340,759-25,841,063). ADAMTS proteases are involved in extracellular matrix proteolysis and are related to morphogenesis, angiogenesis, ovulation, cancer, and arthritis [42]. Due to a lack of biological support, however, we cannot propose *ADAMTSL1* as putative causative gene. The lead SNP on BTA9 (BTA9:80007099, rs382310712) was in a region with no known genes. The region 1 Mb up- and downstream of this SNP contains three genes (*NMBR*, *VTA1*, and *ADGRG6*), all of which are at least 0.6 Mb from the lead SNP. Without additional evidence beyond GWAS results, none of these genes is a likely putative causal gene. Joint analysis picked *TAGAP* (BTA9: 96806294~ 96,815,764) as a candidate gene, but it was too far from the lead SNP (~ 16 Mb; Additional file 1: Table S2). For other QTL, all candidate genes with biological support were located outside QTL intervals.

**Table 4:** Putative causal genes for identified QTL affecting resistance to clinical mastitis

QTL	Gene	Source^*^
3: 91961838~ 93,178,041	*FAM151A*	Gene analysis and RNA-seq
4: 57757451~ 58,741,047	*IMMP2L*	Gene analysis and MP
5: 105444242~ 106,622,012	*FGF6* and/or *CCND2*	Gene analysis and KEGG / Gene analysis, KEGG and nearest gene
6: 23219637~ 23,719,758	*NFKB1*	Gene analysis, KEGG, GO, MP and RNA-seq
6: 88479895~ 88,980,376	*GC*	Gene analysis and MP
6: 92131530~ 93,382,644	*NAAA* and/or *SHROOM3*	RNA-seq and gene analysis / RNA-seq and gene analysis
11: 88150188~ 88,993,125	*ID2*	Gene analysis and MP
13: 61295534~ 62,267,717	*CCM2L* and/or *HCK*	Gene analysis, GO, MP and RNA-seq / Gene analysis, GO, MP and RNA-seq
16: 47048599~ 48,086,099	*HES2* and/or *ACOT7* and/or *KCNAB2*	Gene analysis and RNA-seq / Gene analysis, nearest gene and RNA-seq / Gene analysis and RNA-seq
19: 42148461~ 43,288,858	*KRT32* and/or *DHX58* and/or *KCNH4* and/or *STAT5A* and/or *STAT3*	Gene analysis and RNA-seq / Gene analysis, GO, MP and RNA-seq / Gene analysis, MP and RNA-seq / Gene analysis, nearest gene, KEGG, GO and MP / Gene analysis, KEGG, MP and RNA-seq
20: 38221493~ 38,721,830	*CAPSL* and/or *SPEF2*	Gene analysis and RNA-seq / Gene analysis nearest gene and GO
25: 35104498~ 35,604,430	*ORAI2 / CUX1*	Gene analysis, MP and RNA-seq / Gene analysis, nearest gene and MP

Note, ^*^ we used ‘/’ to separate the evidence for different genes, MP stands for mammalian phenotype database

### Dissecting closely linked QTLs

The low heritability [43] and polygenic nature of mastitis make it challenging to identify putative causal genes associated with variation in mastitis resistance. Several previous studies have reported QTLs for SCC and/or CM, but only one major QTL on BTA6 was identified in several studies [10, 12, 14, 44]. We found 22 independent association signals using imputed WGS variants and CM as a phenotype. Our association analysis approach, which was described previously [27], aids in dissecting closely linked QTLs. The 22 lead SNPs overlapped or were close to many previously reported QTLs [10, 12, 14, 44] and explained 14% of the variance for de-regressed breeding value for resistance to CM.

### Combining multiple sources of information post GWAS

Combining multiple sources of information helped to narrow the list of candidate genes for most QTL. Long-range LD in cattle adds to the challenge of finding putative causal genes in post-GWAS analyses. Here, we combined several independent information sources with GWAS results to short-list the candidate genes. First, we considered physical distance from the lead SNP to the nearest gene, using a similar approach to many previous studies [45, 46]. However, many of the candidate genes (e.g., *NMBR*) had no documented biological function linking them to mastitis, and many lead SNPs may not be located in the causal gene (e.g., due to imputation inaccuracy, random fluctuations in association test statistics, LD structure, multiple closely spaced QTL, etc.). We expected that several variants in a causal gene would show strong associations with the trait. Therefore, we performed gene-based association statistics using MAGMA [17] to test the gene-based association. Still, we cannot draw conclusions based solely on this ranking. Thus, to narrow the candidate gene list further, we applied annotations with GO [24], KEGG and the mammalian phenotype database [26]. To avoid missing candidate genes by ignoring genes outside the list of the top five genes, we included information of DEGs from *E. coli*-challenged cow udders [23] coinciding with gene-based association signals. This joint analysis of gene-based analysis and RNA-seq results helped us to improve information about several candidate genes. For some QTL, however, none of the above approaches led to any candidate gene.

We observed some possible sources of error associated with various approaches of picking candidate genes. For MAGMA, some genes showing significant gene-based association were located very far from lead SNPs. For RNA-seq, we expected to find more immune- or infection-related genes showing differential expression. We checked the distance and LD structure to judge whether to trust the list obtained by joint analysis. For example, *CXCL13*, which is involved in inflammatory and immune responses, was significant in gene-based analysis and was a DEG in the *E. coli* challenge experiment. However, because *CXCL13* is ~ 5 Mb away from the lead SNP, it is highly unlikely to be the causative gene underlying the detected QTL. When the lead SNP was included as a covariate in the second round of GWAS, no additional association signal around this gene was observed. This analysis shows the importance of considering multiple information sources when prioritizing candidate genes for identified QTL. Genes that are not differentially expressed should not be immediately excluded from the pool of potential candidate genes because the sampled tissue or time point might not reflect the place or time of action. Moreover, the causal variant can influence the expression of the other genes that alter the phenotype. We performed variant annotation for all candidate genes. However, it is not possible to design a unique strategy to combine multiple sources of information to pinpoint candidate genes for all scenarios because available information varies from region to region, and differences in modes of gene action can lead to differences in what types of data will be informative. Gradually adding new types of pertinent data and data from other populations will add to the confidence of the identified effects of genes on the variations in mastitis resistance.

### Limitations of the study and potential solutions

With the emergence of new technologies and experimental methods, more data are generated, helping to uncover the biological effector mechanisms of trait-associated genetic variants. However, incomplete genome annotation in cattle still impedes analyses. On BTA9 (BTA9:80007099, rs382310712), we failed to propose any putative causal genes. Poor annotation of this region is probably the main reason for our failure. Three genes have been annotated around 1 Mb from the lead SNP, none of which have functional annotations suggesting them as candidate genes. The inability to identify any genes does not mean that this QTL is a false positive. It may be that the causal mutation is in an unidentified regulatory element or some unidentified gene in the region.

We faced two major limitations in our study. The first concerned the source of RNA-seq data. Although *E. coli*-challenged udder samples can be used to help find genes related to the immune response, mastitis is a complex disease involving multiple pathogens and biological pathways. Ideally, RNA-seq data for mastitis should consider multiple tissues in which these pathways may be active and multiple time points during the development and recovery of mastitis. The second limitation is the long-range LD of the cattle population, which reduces the power of gene-based analysis to discriminate among linked genes. In MAGMA [17], there are two models for different genetic architectures for obtaining the *P*-value after GWAS. The SNP-wise Top (*snp-wise = top*) model is designed for genes with sparse associated SNPs. The SNP-wise Mean (*snp-wise = mean)* model is more attuned to the mean SNP association, but is skewed to high-LD regions. In our experience, the lead SNP is not always the causal SNP [27]. Thus, we wanted to avoid calculating the gene P-value based on the top SNP in genes (*snp-wise = top*), an option that uses only a fraction of the SNPs from each gene. For our purposes, the computational cost of this approach proved excessive. In the end, we used the *snp-wise = mean* option. As a result, the list of genes is biased towards the high-LD region. Long-range LD further lengthens the list of genes with or close to significant SNPs. A possible solution in gene analysis could be implementing meta-analysis from multiple populations to reduce the impact of LD [47] or using a tag SNP strategy [48].

## Conclusions

In this study, we associated 15,552,968 imputed whole-genome sequencing markers with mastitis resistance in 5147 Nordic Holstein cattle. After GWAS, we identified 22 independent QTL that collectively explained 14% of the variance in breeding values for resistance to CM. Besides recovery of some QTL from previous reports, we also found new loci associated with mastitis resistance. To prioritize candidate genes post-GWAS, we used multiple source of information including Gene Ontology terms, Kyoto Encyclopedia of Genes and Genomes pathway analysis, and mammalian phenotype database. To confirm results of gene-based analyses, we used gene expression data from *E. coli*-challenged cow udders from previous study. For combining different information, we designed a method to gradually add new types of pertinent data. At the end, we suggested putative causal genes with biological relevance for 12 QTL affecting resistance to CM in dairy cattle. This study showed our approach could be widely applied for GWAS in dairy cattle.

## Methods

### Procedure to identify putative causal genes

To identify putative causal genes affecting resistance to CM, we linked GWAS results to genes by incorporating biological evidence, as illustrated in Fig. 3. Post-GWAS, we followed two independent approaches to find candidate genes: 1) we used the gene closest to the most significantly associated SNP of the region, and 2) we determined the gene-based association signal strength using MAGMA [17]. For the second approach, we included the top five genes from gene-based analysis because the number of significant genes from gene-based analysis was large.

To move from a list of candidate genes to a list of putative causal genes, we needed biological evidence. We used GO [24], KEGG [25], and mammalian phenotype database [26] as sources for biological evidence with possible terms related to mastitis. Candidate genes with further biological support were accepted as putative causal genes. We also analyzed RNA-seq data from *E. coli*-challenged cattle mammary glands [23]. We confirmed the full set of significant genes from the gene-based association with the DEGs from the RNA-seq data. All candidate genes within a QTL interval that had biological support and were significant in the joint analysis were listed as putative causal genes.

### Phenotype and genotype data

Phenotypic records of CM for Nordic Holstein cattle were obtained from the Nordic Cattle Genetic Evaluation database (NAV, http://www.nordicebv.info/). Values used for association analysis were de-regressed breeding values [49, 50] from the routine genetic evaluation by NAV and were available for 5147 progeny tested Holstein bulls. The association study was carried out on imputed WGS data, as previously described by Iso-Touru et al. [40] and Wu et al. [51]. All bulls were genotyped with the Illumina BovineSNP50 BeadChip (54 k) ver. 1 or 2 (Illumina, San Diego, CA, USA). The 54 k genotypes were imputed to high-density (HD) by IMPUTE2 v2.3.1 [52] with a multibreed reference of 3383 animals (1222 Holsteins, 1326 Nordic Red Dairy Cattle, and 835 Danish Jerseys) with Illumina BovineHD BeadChip genotype data. Next, these imputed HD genotypes were imputed to WGS by Minimac2 [53] with a multibreed reference of 1228 animals from *Run4* of the 1000 Bull Genomes Project [54] (1148 cattle, including 288 individuals from the global Holstein–Friesian population, 56 Nordic Red Dairy Cattle, 61 Jerseys, and 743 cattle from other breeds) [54] and additional data from Aarhus University (80 individuals, including 23 Holsteins, 30 Nordic Red Dairy Cattle, and 27 Danish Jersey) [55]. Imputation to WGS was done in 5-Mb chunks with a buffer region of 0.25 Mb on either side. A total of 22,751,039 bi-allelic variants were present in the imputed sequence data. After excluding SNPs with a minor allele frequency below 1% or with large deviation from Hardy–Weinberg proportions (*P* < 1.0–6), 15,552,968 SNPs on 29 autosomes in Nordic Holstein cattle were retained for association analyses. The average imputation accuracy (*r*^*2*^ values from Minimac2) was 0.85 for cross-breed imputation. Information on the distribution of imputation accuracy as a function of minor allele frequency was published previously [51].

### Methodology of multiple QTL detection and estimation of genetic variants explained by QTL

Details of the association analysis pipeline and simulation verification were reported previously [27]. In the first round, we used GCTA [16] to perform single-SNP GWAS analysis on each chromosome. Subsequently, we fixed the lead SNP as a covariate to run the second round of GWAS if the lead SNP was genome-wide significant (experiment-wise 0.05 type I error-rate after Bonferroni correction for 15,552,968 simultaneous tests corresponds to a threshold of –log_10_P ≈ 8.5). In second and subsequent rounds, if the lead SNP was significant in the first round, we fixed it as another covariate until no more significant association signals were identified. A lead SNP found in any round was tested in 2-Mb flanking regions (up- and downstream) to search for other significant SNPs. If they failed to attain significance, they were removed from further analyses. Remaining SNPs constituted the final list of lead SNPs. To define the boundaries of each QTL region, we examined a 1-Mb flanking region of the lead SNP. If the SNP –log_10_(P) value decreased by more than three units compared to the value of the lead SNP and the region was larger than 0.25 Mb, then we set this SNP as a boundary; otherwise, we set ±0.25 Mb from the lead SNP as the QTL boundary. We used the lead SNP list to generate one genetic relationship matrix (GRM). We removed SNPs within 5 Mb of the lead SNP to construct the second GRM. We estimated the variance explained by the lead SNPs by using GCTA [16] to fit two GRMs.

### LD calculation, variant annotation, and gene annotation

We calculated the pairwise *r*^*2*^ between the lead SNP and all other SNPs on the same chromosome using PLINK [56] and extracted all SNPs with *r*^*2*^ > 0.2 with the lead SNP. These SNPs were annotated by VEP (version 92) [41]. Enrichment of the gene list was estimated by clusterProfiler [35]. Possible candidate genes proposed from different methods were sent to DAVID [57] to retrieve the GO terms [24] of biological process and KEGG pathways [25]. We used the same gene list to search the mammalian phenotype database [26] to check whether mutations in these genes would have some phenotypic effect related to mastitis.

### Confirmation of gene-based association statistics with RNA-seq data

For MAGMA [17], we downloaded the gene information file from Ensembl gene build 92 [58]. The number of genes (including 5′- and 3’-UTRs) with at least one SNP was 20,356; thus, the *P*-value threshold for genome-wide significance was 2.46 × 10^− 6^. A total of 455 Holstein animals from the 1000 Bull Genome Project (*Run 6*) [54, 55] was used as the reference population for MAGMA [17]. We performed MAGMA gene analysis with the GWAS results using the model *snp-wise = mean*. To confirm gene-based analysis results, the list of *P*-values of DEGs from *E. coli*-challenged udders [23] was used. Details of the RNA-seq analysis can be found in a previous study [23]. We only used the list of the DEGs and their false discovery rate (FDR) values. We confirmed the significant genes from gene-based analysis (MAGMA) with the data from the DEGs (RNA-seq).

## Additional file

Additional file 1: **Table S1.** The full list of the significant gene from the gene-based analysis. **Table S2.** The full list of gene significant both in gene-based analysis and differential expression. (XLSX 72 kb)

## Abbreviations

BTA: *Bos taurus* autosome
CM: Clinical mastitis
DEG: Differentially expressed gene
eQTL: Expression quantitative trait locus
FDR: False discovery rate
GO: Gene Ontology
GRM: Genetic relationship matrix
GWAS: Genome-wide association study
KEGG: Kyoto Encyclopedia of Genes and Genomes
LD: Linkage disequilibrium
QTL: Quantitative trait loci
SNP: Single nucleotide polymorphism
SSC: Somatic cell count
WGS: Whole-genome sequence
